# Community Support for Persons with Disabilities in Low- and Middle-Income Countries: A Scoping Review

**DOI:** 10.3390/ijerph19148269

**Published:** 2022-07-06

**Authors:** Xanthe Hunt, Melissa Bradshaw, Steyn Lodewyk Vogel, Alberto Vasquez Encalada, Shanice Eksteen, Marguerite Schneider, Kelly Chunga, Leslie Swartz

**Affiliations:** 1 Institute for Life Course Health Research, Department of Global Health, Faculty of Medicine and Health Sciences, Stellenbosch University, Stellenbosch 7602, South Africa; melissaebradshaw@gmail.com (M.B.); steyn.lvogel@gmail.com (S.L.V.); shanice@sun.ac.za (S.E.); kchungap@gmail.com (K.C.); 2 Center for Inclusive Policy, Washington, DC 20005, USA; alberto.vasquez@inclusive-policy.org; 3 Alan J Flisher Centre for Public Mental Health, Department of Psychiatry and Mental Health, University of Cape Town, Cape Town 7700, South Africa; marguerite.schneider@uct.ac.za; 4 Department of Psychology, Faculty of Humanities, Stellenbosch University, Stellenbosch 7602, South Africa; lswartz@sun.ac.za

**Keywords:** community support, human support, disability, inclusion, participation, LMIC

## Abstract

Over the life course, persons with disabilities require a range of supports to be integrated into their communities, to participate in activities that are meaningful and necessary, and to have access, on an equal basis to persons without disabilities, to community living. We conducted a scoping review of the peer-reviewed and grey literature on community support for persons with disabilities in low- and middle-income countries (LMICs). The main findings of this review concern the following: there are gaps in access to community support for persons with disabilities in LMICs; there are barriers to the provision of such support; formal and informal strategies and interventions for the provision of community support exist across the life cycle and different life domains, but evidence concerning their effectiveness and coverage is limited; and the role of community-based rehabilitation and Organisations of Persons with Disabilities in the assessment of needs for, and the development and provision of, community support, needs to be more clearly articulated. Research needs a more robust theory of change models with a focus on evaluating different aspects of complex interventions to allow for effective community support practices to be identified.

## 1. Introduction

Over the life course, persons with disabilities have a range of support requirements in order to be integrated into their communities, to participate in activities that are meaningful and necessary, and to have access, on an equal basis to persons without disabilities, to community living. These support requirements, some of which are distinctive to persons with disabilities, exist in various life domains, such as communication, decision making, self-care, mobility, and housing (see Table 1 for a non-exhaustive list of examples). These support requirements fall under the umbrella of “community support”, the services and/or assistance necessary to support living and inclusion in the community, and to prevent isolation or segregation from the community, for persons with disabilities [1]. Community support can be provided formally (by governments or the private sector providers) or informally (by unpaid family members or peers). As stated by Vásquez Encalada and colleagues [2] community support “enable[s], connect[s], and leverage[s] accessibility and inclusion efforts by filling the gap between general services and facilities and people’s individual requirements” (p. 5).

The United Nations Convention on the Rights of Persons with Disabilities (UNCRPD) is a human rights instrument codifying the right of all persons with disabilities to their human rights and fundamental freedoms [3]. Usefully, the UNCRPD also explicitly identifies areas where adaptations have to be made to existing systems and practices in order for persons with disabilities to effectively exercise their rights.

The UNCRPD, ratified by 185 States Parties (at time of writing), very explicitly highlights the importance of countries systematically moving from institution-based care, to community-based support [3]. The UNCRPD recognises the right of persons with disabilities to live independently in the community [4]. In order for this community integration to be achieved, a range of supports for community living are required [5] and either implied by or explicitly noted in the Articles [3].

The UNCRPD is also accompanied by an Optional Protocol, which is a side agreement to the main Convention [5]. The Optional Protocol, which has 94 signatories, establishes a complaints mechanism for the Convention, and parties that sign the Protocol agree to recognise the right of the Committee on the Rights of Persons with Disabilities to consider, investigate, and make recommendations in relation to complaints from individuals or groups who claim their rights under the UNCRPD have been violated [5].

Despite this and other international directives to support the rights of persons with disabilities to participate in society and live with dignity, and countries’ responsibilities in relation to them, persons with disabilities experience significant unmet support needs [2,6,7,8,9], particularly in low- and middle-income countries (LMICs). LMICs were classified according to the World Bank lending groups: https://datahelpdesk.worldbank.org/knowledgebase/articles/906519 (accessed on 1 November 2021). In these settings, formal community support services for persons with disabilities are under-developed, under-resourced, or do not exist, leaving persons with disabilities to rely primarily on family members to have their needs met [4,5]. This results in infringements of the rights of persons with disabilities, restrictions in choices, high disability-related costs, and opportunity costs for those providing support [2].

The issue of community support is important to explore, especially because much of the care and support for people with disabilities in LMIC contexts is delivered by informal caregivers and family members, and it is important to understand a wider and more diverse range of networks and resources than are familiar in better-resourced contexts. Where formal support structures exist, providers are commonly overworked, and services are stretched very thin because of lack of funding and availability of personnel specifically trained in community support work relevant to the range of needs of people with disabilities.

Moreover, increasing attention is being paid to the issue of community support, globally. For example, the Human Rights Council’s next annual interactive debate on the rights of persons with disabilities will focus on support systems to ensure community inclusion of persons with disabilities (see A/HRC/49/L.21). Further, the 2022 Global Disability Summit saw several international organisations, including UN entities, donors, governments, and International NGOs, make important commitments to take action toward community inclusion. Commitments include the development, investment, and/or research on community-based support services [10].

Yet, despite this growing momentum, as well as the well-documented needs for support among persons with disabilities and barriers to meaningful inclusion, little literature exists that systematically examines community support practices, especially in LMICs. A comprehensive overview of the current range of evidence on community support is needed as a basis for further planning with due regard to context.

Against this backdrop, we conducted a scoping review of the literature on community support for persons with disabilities with a view to mapping and summarising the evidence about the following in relation to persons with disabilities in LMICs:

1. Gaps in access to community support services;
2. Key barriers to the provision of disability community support services;
3. Formal and informal strategies and interventions for the provision of disability community support across the life cycle and different life domains;
4. Strategies and interventions for community support led by Organisations of Persons with Disabilities (OPDs); and
5. Gaps in knowledge and suggestions for future research.

## 2. Materials and Methods

We conducted a scoping review of the peer-reviewed and grey literature on community support services for persons with disabilities in low- and middle-income countries (LMICs). A scoping review is based on a systematic search of relevant sources but is concerned with different questions to a systematic review. While the latter is well-studied in answering questions about impact and effectiveness, a scoping review is better able to provide a sense of the breadth and depth of a body of research or field. Given the nature of the questions with which this review is concerned, a scoping review was deemed the appropriate methodology. A reference group of six experts on disability in LMICs advised on the research, first through supporting the authors’ conceptualisation of community support, as well as through providing technical comment on the review’s protocol.

### 2.1. Searching of the Peer-Reviewed Literature

The search strategy for the scoping of the peer-reviewed literature was systematic. The search was structured such that multiple databases (including CINAHL, ERIC, Scopus, Web of Science Social Sciences Citation Index, MEDLINE(R), Embase Classic + Embase, PsycINFO, and CAB Global Health) were searched, and supports in multiple domains were identified (including communication, decision making, mobility, assistance with daily living activities, Housing and accommodation, and family support). The peer-reviewed literature search was run in English and French.

Given the diversity of domains proposed relevant to community support, the search strategy included a wide variety of intervention terms. The population and location terms were based on recent Campbell Collaboration search strategies for evidence gap maps (EGMs) on disability inclusive development in LMICs. The full search strategy and a record of all searches are available from the authors upon request.

### 2.2. Searching of the Grey Literature

The second stream of evidence mapping that informed our scoping was a review of the grey literature on community support services for persons with disabilities in LMICs. For this process, key websites (such as those of UNICEF, the World Bank, the International Labour Organization, SightSavers, CMB, etc.) were searched for relevant reports, briefs, and other grey literature. The grey literature searches were run in English, French, and Spanish. The full grey literature search strategy and a record of all searches are available from the authors upon request.

### 2.3. Analysis

Abstracts and full texts of identified publications were screened using Rayyan.ai. All abstracts and full texts were double screened by M.B., S.E., and S.L.V., and conflicts were resolved by X.H. Reference lists of reviews identified by the search were examined for eligible publications. Included publications were coded according to publication type, allowing for each included publication to be organised as either quantitative or qualitative, and according to whether it presented descriptive data (for instance case descriptions, needs assessment findings, survey results, etc.) or documented practices (such as impact evaluations, best practice guidelines, etc.).

Once this process was completed, each included study had standardised information systematically extracted from it according to a coding sheet. The coding sheet was piloted with 5 studies prior to use on all included papers. The coding sheet differed for qualitative and quantitative literature, and for descriptive studies and impact evaluations. However, it included domains such as:

1. Target population impairment type;
2. Country;
3. Ecological level (individual, family, community, or policy and environment).

A short narrative summary of documents was also produced. The grey literature was sorted into high- and low-priority items (based on the specificity of the content to the questions of this review), and the focus of the data synthesis described below was on the high-priority items.

### 2.4. Synthesis

Once coding and extraction was complete for both bodies of literature, the data were examined, and overarching topics and themes identified. These topics and themes were then narratively synthesised with the aim of answering the key questions guiding this review.

## 3. Results

**Figure 1:**
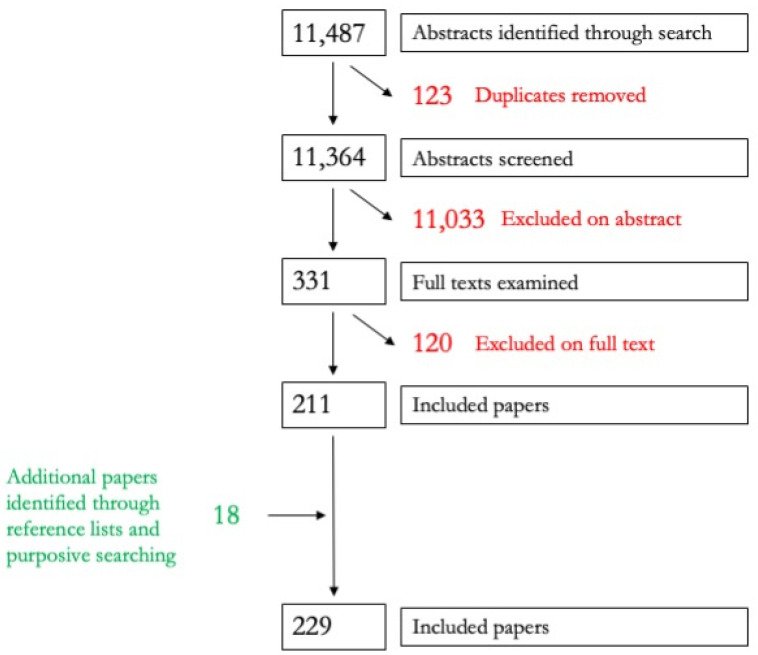
PRISMA flow chart for the peer-reviewed literature.

**Figure 2:**
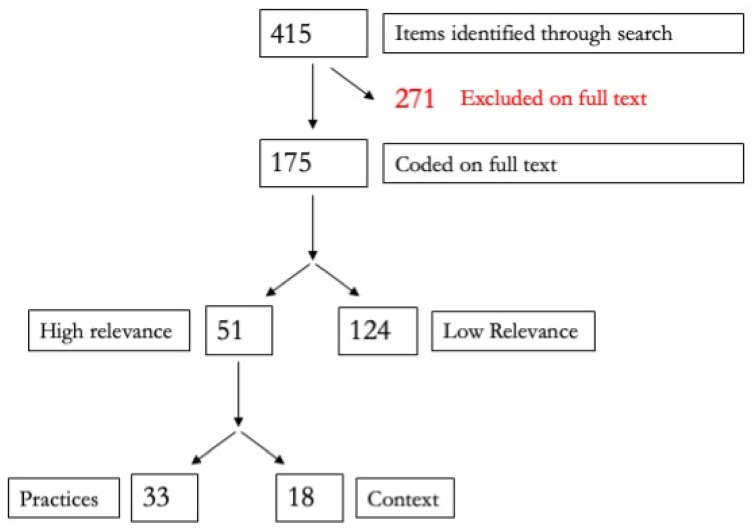
PRISMA flow chart for the grey literature.

The results of the search and screening processes for both bodies of literature are presented in the PRISMA flow charts at Figure 1 and Figure 2.

Our final pool of included literature comprised 229 peer-reviewed studies and 175 grey literature items. Within the peer-reviewed literature, the preponderance of literature presented qualitative data. Of the 229 included papers, there were 143 presenting qualitative data, 54 presenting quantitative data, 13 reporting on mixed-methods research, and 19 reviews. The overwhelming majority of peer-reviewed papers were also descriptive in nature, with 187 studies presenting descriptive data and [11] reporting on impact evaluations. The majority of the literature was concerned with people with hearing impairments, cerebral palsy, and physical impairments., with other impairment types less represented.

Forty-eight LMICs were represented in the peer-reviewed literature, with South Africa, India, China, Brazil, and Ghana each contributing over 10 peer-reviewed papers each.

Within the grey literature, there were 166 presenting qualitative data, 3 presenting quantitative data, and 6 reporting on mixed-methods research. The overwhelming majority of papers were also descriptive in nature, with 171 items presenting descriptive data and 4 reporting on impact evaluations. Forty-eight countries were covered in the grey literature, with many publications reporting on whole regions or multiple countries.

Of the 48 LMICs identified in this scoping review, all are signatories to the CRPD, but only 28 of them are signatories to the Optional Protocol.

**Figure 3:**
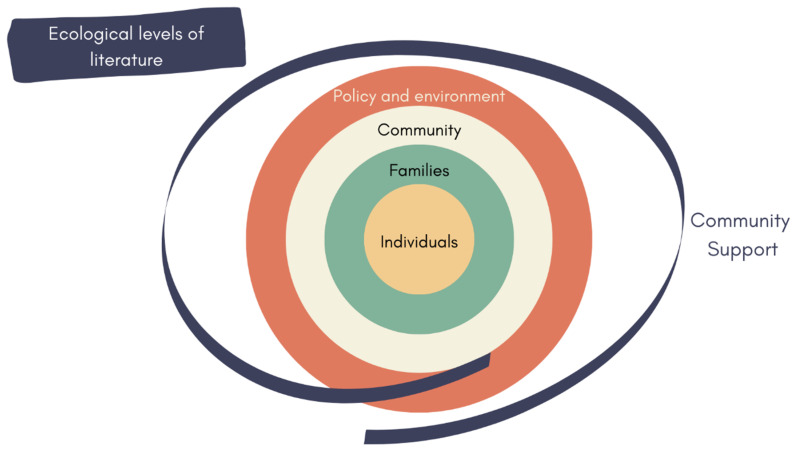
Ecological model according to which literature was organised.

The included literature yielded insights that were usually either of primary relevance to thinking about persons with disabilities themselves, their families and carers, the wider communities in which they live, and then the societies and environments in which those communities are embedded. We use this ecological framework (see Figure 3) to discuss our findings in the sections that follow.

**Figure 4:**
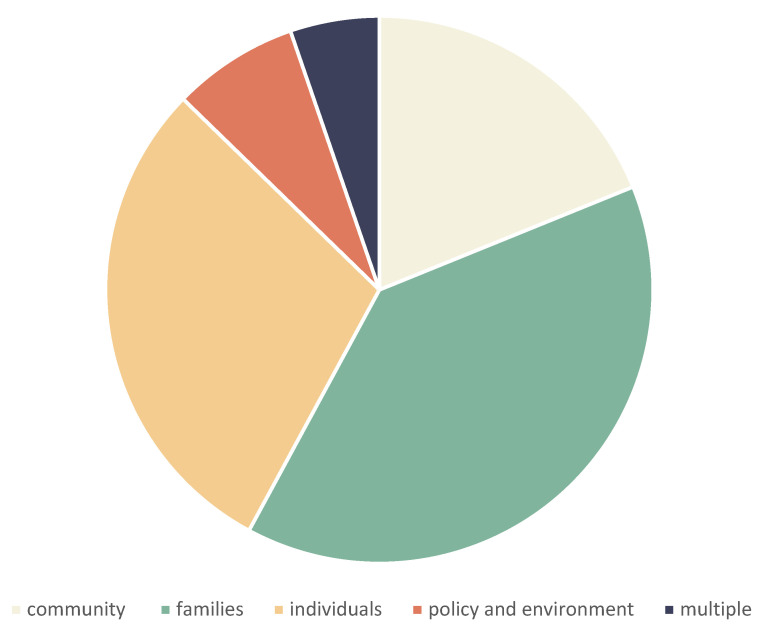
Peer-reviewed literature according to ecological level.

The ecological level most frequently represented in the peer-reviewed literature was family, followed by individual. The minority of studies concerned the community and policy and environmental levels. This is shown in Figure 4.

**Figure 5:**
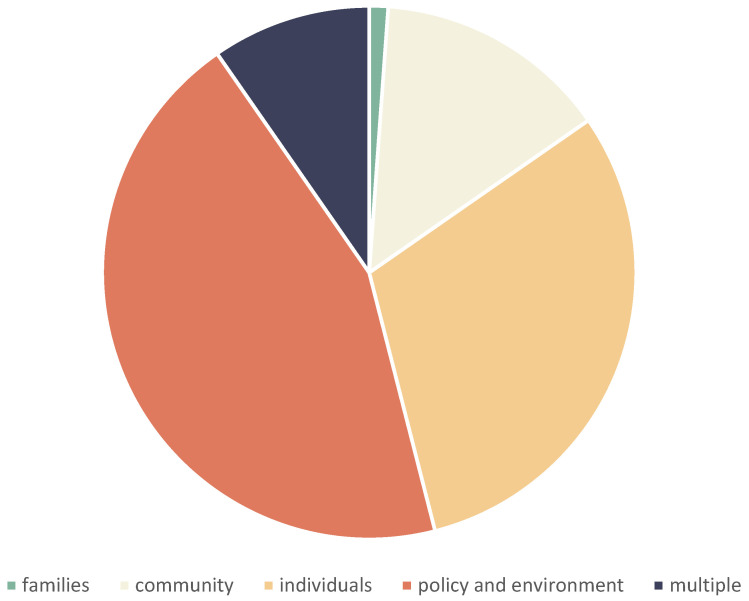
Grey literature according to ecological level.

Unlike the peer-reviewed literature, however, the vast minority of the grey literature concerned the higher ecological levels, and the majority concerned the policy and environment level: there were only 2 items concerning families, 25 on communities, 54 on individuals, 17 covering multiple domains, but 78 concerning the policy and environment level (see Figure 5).

**Figure 6:**
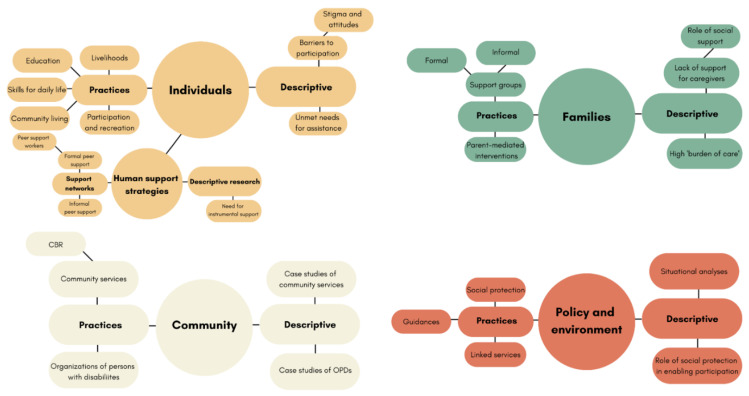
Mapping of key topics in the literature.

Key topics within each ecological level are presented in Table 2 (below) and mapped in Figure 6. Cross-cutting themes included: the life course, definition of community support, and quality of evidence.

## 4. Limitations

While comprehensive, this scoping review has some limitations. Firstly, while we cover three languages, there could be important literature—most likely grey literature—published in Russian, Arabic, Portuguese, Swahili, and a range of other languages. Secondly, there are likely many more practices than those identified, but these have not been formally documented. It is also possible that, given the breadth of the topic and the wide range of fields concerned with community support for persons with disabilities, some literature may have been missed by our search.

## 5. Discussion

### 5.1. Individual Level

When it comes to community support, the peer-reviewed literature is disproportionately concerned with showcasing the need for community support, documenting barriers to participation, and generally describing the ways in which community support services could contribute to the wellbeing of persons with disabilities (This review is not concerned with barriers to accessing healthcare services; however, these are numerous. They have been well documented elsewhere (see e.g., Bright, T., Wallace, S., & Kuper, H. (2018) [12]. A systematic review of access to rehabilitation for people with disabilities in low- and middle-income countries. International Journal of Environmental Research and Public Health, 15(10), 2165). However, it lacks examples of evaluation of specific support practices. The grey literature also contains extensive documentation of barriers to participation and services access and needs among persons with disabilities. However, it also documents a number of promising community support practices. Unfortunately, in most cases, there was too little detail in these documents to get a firm understanding of the dosage, implementation, scale, or other specifics of practices.

Topics covered in this literature included barriers to participation [7,13,14,15,16,17,18,19], unmet needs for assistance [7,8,9,20,21,22,23,24], human support and peer support [25,26,27,28], and documentation of specific programmes [26,29,30,31].

This literature shows that gaps in access to community support are well documented: persons with disabilities in LMICs have a range of unmet needs in relation to accessing formal services, getting disability-related information, developing strategies for daily living, receiving support for childcare among parents of children with disabilities, accessing social support and other social resources, finding support for communication and socialisation, and being able to pursue employment opportunities in line with personal preferences.

A subsection of the literature on needs among persons with disabilities was concerned specifically with unmet needs for assistance [5,13]. For instance, Andrade and Andrade [7] showed that older persons with disabilities have a high proportion of unmet needs, both in the basic activities of daily life (such as bathing, dressing, walking, using the toilet, feeding, and moving in and out of bed), as well as the instrumental activities of daily life (such as managing finances, using transportation, shopping, and taking medication). In papers from Thailand [21] and China [22,23,24], specific unmet needs are highlighted:

- Access to available and continuous rehabilitation services local to where persons with disabilities live;
- State welfare or other financial support;
- Information for parents of children with disabilities about disability and how to provide care and opportunities for their child;
- Support for childcare among parents of children with disabilities;
- Social support and other social resources;
- Support for activities required for inclusion in society (e.g., communicating, doing housework, working outside, making friends); and
- Support to pursue employment opportunities in line with personal preferences.

A study from China, reported by Wu et al. [22], characterised unmet needs for elderly care—ADL and IADL—in a sample of community-dwelling older persons with disabilities, and provided a useful amount of detail for understanding how unmet needs influence persons with disabilities’ daily lives. Importantly, the study found that older persons with disabilities had a higher number of unmet needs with regard to both ADL and IADL assistance, largely due to a lack of linkages to care resources when compared to older persons without disabilities. Factors that facilitated linkages to care included independence (e.g., mobility), interpersonal factors (e.g., the number of close children and living arrangement), and social environment (e.g., neighbourhood type). These resources for linkage to care, and not only the specific availability of care resources in the environment, were identified as important in meeting the care needs among older persons with disabilities. This study highlights the significance of the role of the person—environmental linkage in reducing the unmet needs for ADL and IADL assistance among persons with disabilities, and highlights the role that community support services could play in linking persons with disabilities to available services.

At the level of the individual, the grey literature documenting ‘practices’ (akin to the impact evaluations or interventional studies of the peer-reviewed literature) is dominated by reports and press releases from International Non-Governmental Organisations (INGOs), funding bodies, and other stakeholders, sharing case studies on specific community support initiatives [32,33].

Unfortunately, in most cases, there is too little detail in these documents to get a firm understanding of the dosage, implementation, scale, or other specifics of practices.

This is despite the fact that several promising contributions to community support were reported, including, for instance, the deployment of sign language interpreters to communicate with local officials, courts, teachers, and doctors in Serbia, or the provision—by the Dominican Republic Social Protection Program—of care for children with disabilities so that their caregivers can engage in labour market activities [34]. In the example from the Dominican Republic, a social protection programme supported by the World Bank [21], the Dominican Republic Social Protection Program, provides care for children with disabilities to enable caregivers to work or further their studies. In one example shared in the grey literature, a mother was able to attend classes at a vocational training centre whilst her children were taken care of at a day care. Numerous other innovative programmes are reported in the grey literature, but formal evaluations are not readily identifiable. These include USAID’s New Justice activity, which sees courts connected with organisations that represent persons with hearing impairments to increase understanding and cooperation on the procedures for hiring interpreters in Ukraine [35] and livelihood programmes such as inclusive digitally supported farming [36].

The individual-level literature also contained much on the use and roles of peer support. An important note to be made here is that our review is chiefly concerned with community support as an intervention in and of itself. This is a subsection of the much broader literature documenting how peer support can be utilised as a mode of delivery for other programming. While community support might be achieved through both (through peer support as an intervention, and through other programming delivered via peer support networks), we are more interested in this review in the former. This is because peer support for the delivery of programming can be utilised to deliver a whole range of programmatic content that may not be aimed at supporting community inclusion and participation.

Some examples of relevant types of programmes include Janagama, Addanki, and Lingutla’s [29] examination of stroke support group meetings in helping stroke survivors and care givers. This literature also shows how formal programming in a range of domains can serve as a springboard for the development of informal networks for the provision of community support. Another example of such peer support as intervention programmes is in the use of a Chinese OPD’s service user network being used to create a social media support system for persons with physical disabilities [37].

In the grey literature, there was also a great deal of documentation of the form and functioning of self-help groups, including in Asia and the Pacific [38] and in Tibet [39]. As far as these groups are concerned, the grey literature primarily presents case studies showcasing how self-help organisations can advocate for the rights of persons with disabilities at the local level, and offer opportunities for instrumental support among persons with disabilities in a given location.

Two important findings from the literature on support groups and peer support networks include that, where support groups are not disability-targeted—for instance women’s groups or parenting groups with membership of persons with and without disabilities—there may be barriers to participation for persons with disabilities, and so targeted initiatives may be needed [27]. Secondly, where support networks are seeking to leverage technology to expand access, inequalities in terms of access to mobile technologies creates a risk for widening inequalities [28].

While it appears that peer support is an important component of interventions to achieve community inclusion for persons with disabilities in LMICs, it is unclear whether these networks function best when they develop organically, or whether there is a place for those delivered in a top-down model (as programmatic elements of NGO interventions). The former may lack resources, and the latter may not reflect the priorities of persons with disabilities.

Overall, the publications identified by this review concerning the individual level show that, while needs among persons with disabilities are well documented, these are not commonly articulated as a set of priorities for community support. Despite a significant focus on community inclusion and community participation in research and advocacy documents, there is no well-developed literature concerning community support, what it means, and why it is important for community inclusion.

Importantly, human support is not extensively studied in the peer-reviewed literature, likely due to the difficulty in isolating core components of human support, which often arises organically and exists on a small scale and operationalising them in the contexts of a study. While the grey literature contains examples of human support strategies [21,36,38,39], too little detail is recorded in most grey literature documents to allow for the strategies’ impact to be assessed.

### 5.2. Family Level

A significant amount of the literature documenting gaps in access to community support services for persons with disabilities, barriers to the provision of disability community support services, and general challenges in achieving participation and inclusion for persons with disabilities in LMICs is concerned with caregivers. This is likely because, in LMICs, the vast majority of carers are family members (in one study, the proportion of carers who were family members was 94.3% [40]). The challenges experienced by primary caregivers of children with disabilities are well documented [41,42], and a high ‘burden of care’ (the authors prefer not to utilise the term ‘burden of care’, as it can be seen to imply that people with disabilities are burdensome, which is erroneous. However, the terminology is utilised extensively in the field, and given that this is a review, where reporting on primary studies that utilise this language, we replicate it.) among other family carers who are not parents is also reported [6,43,44]. As such, the literature concerning the family level could be broadly divided into two topics: needs among caregivers and caregiver programmes.

The literature seems to suggest that programmes for caregivers, particularly parents, could be a valuable part of community support because they could support parents or other caregivers to facilitate persons with disabilities’ participation. However, those seen in the peer-reviewed literature are often medicalised, focus quite narrowly on ‘management’ of persons with disabilities, or are focussed caregivers’ coping and not persons with disabilities’ participation [45,46]. While these areas of intervention are important and warrant programmatic effort, caregiving programmes could be being leveraged to enable participation and support empowerment of persons with disabilities, and it is unclear to what degree this is being done.

Overall, at the family level, the literature clearly articulates needs among caregivers. However, there do not appear to be many programmes for caregivers that look at persons with disabilities’ participation as a key outcome.

### 5.3. Community Level

The literature concerned with needs and supports at the community level was chiefly concerned with either the implementation and documentation of community-based programming, including CBR, or the form and functioning of Organisations of Persons with Disabilities (OPDs). Topics covered by the literature concerned with this level included the tension between community-based rehabilitation as location and community-based rehabilitation as a holistic strategy [1,47,48,49,50,51,52,53,54,55], and the role of OPDs in community support.

Regarding CBR, there appears to be a tension between CBR as place and CBR as strategy. CBR, when seen in the context of task-shifting in the health system, does not necessarily constitute an avenue for community support. However, where CBR is seen as a model of delivering a holistic package of services and supports to persons with disabilities in the communities where they live, then the opportunities to deliver community support through CBR infrastructure are numerous. The literature on CBR reflected both perspectives, but it was not clear whether the opportunities of ‘CBR as strategy’ were being capitalised on for community support. An understanding of the relational, as opposed to the task-oriented aspects of CBR, is key in this regard. In a number of cases, outcomes of programmes are reported rather narrowly; the assessment of social change is difficult but needs to be considered as the field develops.

This body of work also shows that, despite a significant focus on community inclusion and community participation, there is no well-developed literature concerning community support, what it means, and why it is important for community inclusion. The role to be played by community-based rehabilitation, including which entry points it provides for community support and how these may be leveraged, is unclear.

While OPDs have a valuable role to play in the lives of persons with disabilities in LMICs [37,56], and they require access to sustainable funding to function optimally and be resilient to shocks, their precise role in the assessment of needs for, and the development and provision of, community support, remains unclear.

### 5.4. Policy and Environment Level

Peer-reviewed literature on policy responses to the need for community support is lacking. The grey literature offers more in terms of information relevant to understanding the policy provisions and enabling environments necessary for the provision of community support than does the peer-reviewed literature. This may be in part due to the difficulty of formally evaluating policy for the purposes of publication, and the preponderance, in this grey literature of situational analyses [57] and frameworks for action and technical guidance. Topics within this level of literature included social protection and policy and systems [9,11].

While there are examples of OPDs being involved in the planning or government responses for persons with disabilities [58], this appears to be more often in relation to formal services’ development such as rehabilitation or education than community support.

A fair amount of the peer-received and grey literature at this level concerns the role of social protection in community support. In several countries, the provision of non-contributory cash transfers can be seen as promoting community support. For example, in Cambodia [58], the social protection system is underpinned by the involvement of OPDs, which use the social protection system as a vehicle for awareness-raising and inclusion campaigns. In other settings, more comprehensive social protection programmes are being developed, incorporating not only grants/stipends/cash allowances/pensions, but a host of linked services.

Thailand and Tunisia are developing personal assistance service schemes [59], which have the potential to link individuals to social networks. Cash transfers delivered on mobile platforms, such as those being developed in Gambia, Morocco, and Togo, have the potential seamlessly to link beneficiaries to other sources of support [60]. In Vietnam, applications for all forms of social protection are conducted at the local commune-level People’s Committees, one of the lowest administrative units, and automatically linking beneficiaries to local infrastructure, and a shift from a previous, more centralised, system. Research shows that this decentralised approach has been seen to improve coverage under disability-targeted programmes [61,62]. This may relate as well to a move in Vietnam to the use of functioning-based protocols as opposed to purely medical-based assessments [61]. In a shift from previous policy, all Cambodians who qualify for disability cash transfers now automatically qualify for free health insurance, a move that may also increase participation by persons with disabilities in society [58].

An important learning from the grey social protection literature, as far as community support is concerned, is that where government supports include non-financial benefits, such as transport subsidies, educational stipends, and other linked services and benefits, it is imperative that these are accessible at point of use for persons with disabilities. Persons with disabilities also need to be made aware of the full range of benefits available to them, as low uptake hinders the ability of such provisions to meaningfully impact the participation of persons with disabilities [63].

In terms of social protection, one particularly striking feature of the grey literature is widespread reports of cash transfers, vouchers, and other forms of conditional and unconditional social protection [64], including in Syria [65]. While these provisions can be thought of as existing at the same level as policy, in terms of being important conditions for the creation of enabling environments for persons with disabilities, it is also worth noting that many informal social protection initiatives, such as savings and loans groups, have been documented at the community level, including in Uganda [66]. The precise role of these latter networks in providing material resources for community support among persons with disabilities could usefully be evaluated more explicitly.

Going forward, evaluations of social protection and other cash transfer programmes should look specifically at outcomes relevant to community support and also see allocation of funds within a cash transfer on community support. Furthermore, it is not clear whether services linked to social protection programmes map onto the self-identified community support needs of persons with disabilities and are accessible and usable, and if they are not, what strategies can be used to strengthen them.

Longer-term evaluations that assess the robustness and longevity of programmes designed to promote inclusion are key. Mechanisms for evaluating the manner in which social protection, including small, informal, community networks such as savings and loans groups, in terms of key outcomes of community integration and participation, are needed. There is a lack of peer-reviewed literature at this level relevant to thinking about community support for persons with disabilities, or documenting policy responses on community support beyond cash transfers.

### 5.5. Cross-Cutting Issues

Most of the peer-reviewed literature is concerned with adults, including adults with disabilities and adult caregivers of children with disabilities. Specific literature concerning the priorities of young persons and the elderly does exist [6,8], but is limited in comparison to the work conducted with adults. The grey literature showcases examples from a wider spectrum of populations and better represents the life course, with equal numbers of publications concerning children, and adults, with disabilities.

The lack of consensus definitions of what community support is means that synthesising evidence on it is difficult. A clear definition would allow for the identification of key actors (who needs community support, who might be providing it) and the clear examination of interventions and evaluation of the effectiveness.

The grey literature contains many examples of the kinds of community support in which this review is interested [21,32,33,35,36,37,38,39]. However, these are largely small anecdotes or press-release case studies, and so much about the interventions in question is not possible to discern.

### 5.6. Key Findings

The main findings of this review concern the following: there are gaps in access to community support for persons with disabilities in LMICs; there are clear, identifiable barriers to the provision of such support; formal and informal strategies and interventions for the provision of disability community support exist across the life cycle and different life domains, but evidence concerning their effectiveness and coverage is limited; and the role of community-based rehabilitation and Organisations of Persons with Disabilities in the assessment of needs for, and the development and provision of, community support, needs to be more clearly articulated.

Gaps in access to community support are well documented. Persons with disabilities in LMICs have a range of unmet needs in relation to accessing formal services, getting disability-related information, developing strategies for daily living, receiving support for childcare among parents of children with disabilities, accessing social support and other social resources, finding support for communication and socialisation, and being able to pursue employment opportunities in line with personal preferences. A major unmet need is for linkages between persons with disabilities in the community and available resources. Part of the difficulty in studying community support is that though it is often an implicit goal of projects, this is not often formally evaluated.

Regarding barriers, the main barrier that this review can identify to the provision of community support to persons with disabilities is that lack of a formal agenda, framework, or plan for articulating community support priorities and developing and evaluating programming in line with these. Other barriers relate to resources, to the lack of disability mainstreaming, to the challenges of local infrastructure, and to the bias in social programming to shorter-term projects and evaluations.

Formal strategies for community support include community-based programming. Community-based rehabilitation (CBR) is one formal vehicle of community support in cases where CBR is operationalised as a holistic strategy rather than simply as decentralised provision of traditional services. Many other formal strategies are documented (largely in the grey literature), but the evidence is thin, and rigorous documentation of implementation practices, scale, and impact are lacking. Informal strategies, similarly, are not well documented.

The role of OPDs in providing community support includes leveraging their membership to disseminate information and create social and peer support networks; providing input into policy and planning, particularly in respect of social protection and linked services; delivering small-scale community-based support programmes; and providing a platform for persons with disabilities to engage in conversations with other key stakeholders when community support services are being developed. However, while OPDs have a valuable role to play in the lives of persons with disabilities in LMICs, their precise role in the assessment of needs for, and the development and provision of, community support, remains unclear.

## 6. Conclusions

An overarching learning is that community support is a topic around which various bodies of evidence orbit, but upon which few studies specifically land. Further, instrumental support (human support) practices do not seem to be well-captured in the peer-reviewed literature, most likely due to their informality. Indeed, community support practices are informal and not easily isolatable as a variable. Activities that constitute community support are often linked to other activities.

Such practices are captured to a greater degree in the grey literature. However, our degree of confidence in learnings documented in this type of literature is limited. Nonetheless, it should not be the case that more rigorous evidence is taken to be a precondition for investment in community support: for the reasons noted above, the very practices that constitute community support may not be readily amenable to evaluation in this manner. Instead, methodologies are required to synthesise learnings from small-scale studies and the grey literature, as well as potentially from experts’ accounts, so that good practice recommendations can be made.

This review constitutes one step towards this goal, and the implications of our synthesis for practice have been summarised above. It remains, then, to briefly comment on implications for research: research needs a more robust theory of change models with a focus on evaluating different aspects of complex interventions. There must be a balance between smaller-scale local research with a specific focus and more programmatic interventions and assessments. The issue of sustainability of change is not well covered in the literature and is a key gap; this has implications for funding models for research.

Further, to move the peer-reviewed literature forward, a consensus definition of community support needs to be arrived at, and its key outcomes must be operationalised. This is currently absent from the literature, and indeed posed challenges for this review, as the broad scope of what can be considered community support under the various definitions makes homing in on relevant literature difficult. This will allow for actions and commitments made to be monitored, evidence-based practices to be developed, and stakeholders held accountable.

Finally, methodologies are required to allow for the synthesis of learnings from small-scale studies and from the grey literature, as well as potentially from experts’ accounts, so that good practice recommendations can be made. Flexible methodologies drawing on multiple sources of information, such as those utilised in this review, but potentially even more extensive in scope, are needed.

## Figures and Tables

**Table 1:** Examples of domains of support.

Example Domain of Support	Description	Examples
**Communication**	Support to overcome barriers that limit the ability to communicate and be understood.	e.g., sign language interpretation, tactile interpretation
**Decision making**	Support to make decisions and exercise legal capacity.	e.g., support agreements, peer support, self-advocacy support
**Mobility**	Support for personal mobility and access to affordable and available quality mobility assistance.	e.g., assistance animals, point-to-point transport *For this review, assistive devices are not included.*
**Assistance with daily living activities**	Support to assist persons with disabilities in a one-to-one relationship to perform daily life activities, including Activities of Daily Living (ADL) and Instrumental Activities of Daily Living (IADL).	e.g., full or part-time professional personal assistance, third-person support allowance
**Housing and accommodation**	Support with housing and living arrangements, including home modifications.	e.g., housing information and assistance, home support, supported living services
**Family support**	Support to families and persons with disabilities around family, informal support for persons with disabilities, community engagement	e.g., awareness-raising, peer support groups

**Table credit:** Reprinted/adapted from Vásquez Encalada, A.; Gupta, S.; Cote, A.; Tanhchareun, T.; Ghanem, A.; Pereira, M. A. (2021). The Disability Support Gap: Community support for persons with disabilities in low- and middle-income countries. Discussion paper. Centre for Inclusive Policy.

**Table 2:** Key topics covered in the literature.

Ecological Level	Topic
**Individual level**	Barriers to participation
	Unmet needs for assistance
	Human support and peer support
	Documentation of specific programmes
**Family level**	Needs among caregivers
	Caregiver programmes
**Community level**	Community-based rehabilitation as location
	Community-based rehabilitation as holistic strategy role of OPDs
**Policy and environmental level**	Social protection
	Policy and systems

## Data Availability

The data for this review (.RIS files, Excel files) are available from the authors upon request.
